# Hi-fun, homepas and incidental sex with drugs: a qualitative study developing a locally grounded definition of hi-fun (chemsex) compared to other sexualised drug use types practiced by gay, bisexual and other men who have sex with men in Thailand

**DOI:** 10.21203/rs.3.rs-7211745/v1

**Published:** 2025-07-29

**Authors:** T Charles Witzel, Worawalan Waratworawan, Nattharat Samoh, Alison J Rodger, Harry Prabowo, Gloria Lai, Pongsri Bootsan, Ittiphol Chaicharoen, Thissadee Sawangying, Ratachayapornthawee Thanawattewakul, Siriporn Nonenoy, Sudarat Thongsuksangcharoen, Nittaya Phanuphak, Siripong Srichau, Verapun Ngamee, Adam Bourne, Thomas E. Guadamuz

**Affiliations:** Mahidol University Faculty of Tropical Medicine; Mahidol University Faculty of Tropical Medicine; Mahidol University Faculty of Tropical Medicine; University College London; APN+; International Drug Policy Consortium; ACTTEAM; ACTTEAM; Health and Opportunity Network; Health and Opportunity Network; Institute of HIV Research and Innovation; Institute of HIV Research and Innovation; Institute of HIV Research and Innovation; APCOM Foundation; Ozone Foundation; La Trobe University Melbourne; Mahidol University Faculty of Tropical Medicine

**Keywords:** chemsex, sexualised drug use, gay, bisexual and other men who have sex with men, methamphetamine, stimulant drugs, criminalisation, Thailand, Southeast Asia, globalisation, transnational queer sociology

## Abstract

**Background:**

Gay, bisexual and other men-who-have-sex-with-men’s (GBMSM) sexualised drug use (SDU), defined as taking psychoactive drugs before or during sex, is the focus of public health concern globally. ‘*Hi-fun*’in Thailand (similar to the practice of ‘chemsex’ in Western settings) is a subset of SDU. Much harm reduction programming relies on Western chemsex definitions, obscuring critical variation related to local cultures, drug markets and legislative contexts. We aimed to develop, informed by transnational queer sociology, a locally grounded definition of *hi-fun* compared to other SDU types among GBMSM in Thailand.

**Methods:**

To delineate SDU types and explore structural and social influences on how hi-funis practiced and organised, focus groups and in-depth interviews (April-Sept 2024) were conducted with GBMSM (with SDU experience within 12-months) recruited from community organisations in Bangkok, Khon Kaen and Pattaya. Data were transcribed, translated and analysed with a thematic framework.

**Results:**

Participants (n=30) were aged 25–47 years, 25 gay, ten with HIV, seven born outside Thailand. Most (n=23) used crystal methamphetamine (*ice*) before/during sex in preceding 12-months, with fewer taking other drugs (ecstasy/MDMA=14, ketamine=12, cocaine=10, GHB/GBL=5). Participants’ accounts coalesced around three main SDU types: *hi-fun*, sex at *homepas*, and incidental sex with drugs.

*Hi-fun* was delineated from other SDU types based on participant motivations to increase wellbeing through pleasure and intimacy, in contrast to homepas and incidental sex with drugs which were linked more to socialising. Crystal methamphetamine (*ice*) was considered foundational to *hi-fun*, whereas other drugs (e.g. cocaine, ecstasy/MDMA, ketamine and GHB/GBL) were more common in *homepas* and incidental sex with drugs. Technology, especially geolocation social/sexual networking apps, were central to *hi-fun*organisation, but potentially less important for other SDU types. Both *hi-fun* and *homepas* primarily took place in private settings, whereas incidental sex with drugs happened in a wider range of venues.

**Conclusions:**

*Hi-fun* in Thailand can be defined as the intentional combination of sex and crystal methamphetamine (ice) to enhance intimacy and pleasure with one or more other man/men, facilitated by technology and usually in a private setting. This definition will be useful for those supporting GBMSM in Thailand through policy, research and service provision.

## Background

Gay, bisexual and other men who have sex with men’s (GBMSM) sexualised drug use, defined as taking psychoactive drugs before or during sex, is the focus of much public health concern globally (1). Research in this area originated in high-income Western countries (primarily in Europe and North America) around a decade ago (1–8), however in the last five years GBMSM sexualised drug use has increasingly been the focus of both public health attention and academic scholarship in East and Southeast Asia (9–14), with an emerging evidence base in Thailand (15–18). A recent systematic review found that between 11.1–17.9% of GBMSM in Thailand reported lifetime engagement in sexualised drug use, although definitions of what constitutes sexualised drug use varied across studies, complicating prevalence estimates (19).

‘*Hi-fun*’ in Thailand, or ‘chemsex’ in many Western settings, is a subset of GBMSM sexualised drug use. Chemsex is typically defined as the use of specific drugs (e.g. methamphetamine, GHB/GBL, mephedrone) to intensify and prolong sex, often with multiple partners (3). This definition emerged from a study focussed on a small geographical area of South London in 2013, one which holds a large population of gay and bisexual men (5, 6). It has largely dominated academic and service delivery understandings of chemsex globally, however there has been a considerable lack of attention as to how local factors shape practices, cultures and understandings of this type of sexualised drug use in many settings. For example, diverse drug markets, differing economic and political contexts within and between countries, wider cultural influences and diverse queer identities will all have a substantial impact on how such drug use is conceptualised and understood, shaping both sexualised drug use practices and potential health outcomes (20, 21). Relying on definitions of sexualised drug use types emerging from Western countries in settings where they do not reflect local realities also risks the development of research and programming which is inattentive to the needs of the groups they seek to serve. Indeed, there is growing focus on the need to establish regionally specific definitions of chemsex/*hi-fun* (22). Developing more locally grounded understandings of variations around sexualised drug use is therefore critical to support effective research and service provision.

A further issue is a lack of focus on the diversity of sexualised drug use *types* that GBMSM practice globally. Much research around GBMSM sexualised drug use focuses solely on chemsex as it is perceived to be potentially more harmful than other forms because of links to longer durations of sex with a greater number of partners, and the involvement of drugs linked to potentially worse health impacts (23). This is compounded by a tendency by some to define chemsex so broadly that many diverse behaviours are incorporated (1, 17, 22), despite communities engaged likely having much more nuanced views of the meanings and motivations underpinning various sexualised drug use practices. Indeed, pilot research conducted with key informants in Thailand in support of this study found that boundaries between *hi-fun* and other sexualised drug use types were not well defined amongst service providers: concern was expressed about responding to a range of substances/behaviours including, and beyond *hi-fun* (20), highlighting the critical need for a locally grounded definition of *hi-fun* compared to other types of sexualised drug use.

Sexualised drug use in Thailand is shaped by the unique social, political, economic and cultural forces of the country. Firstly, Thailand faces intense wealth inequality, and has been described as one of the most unequal countries in the world (24, 25). This has unknown impacts on how sexualised drug use is practiced, although in Thailand *hi-fun* is normatively associated with wealthy, Westernised GBMSM and new hierarchies appear to emerge in *hi-fun* spaces (15, 20). Secondly, the legislative environment likely has a substantial influence on sexualised drug use practices as illicit substance use is intensely criminalised in Thailand. Possession of drugs is often considered trafficking, with exemptions for very small amounts of substances, in which cases police officers can refer individuals to forced rehabilitation programmes (26, 27). There are reports of the police entrapping and extorting people who use drugs, including GBMSM engaged in sexualised drug use (20). How these encounters with state actors influence the ways in which *hi-fun* is organised and practiced is unclear. Thirdly, although Thailand is often conceptualised as ethnically and culturally homogenous, it is a major centre of gay life in Southeast Asia attracting huge volumes of sexual and gender minority tourists, and substantial numbers of migrant GBMSM from other Southeast Asian countries and further afield call Thailand home (28–30). As a consequence, local and culturally-specific practices surrounding sex and drug use in many parts of the world are brought to Thailand and likely interact with one another. This has an unknown impact on how sexualised drug use is practiced and defined in this context.

### Theoretical grounding

We used Transnational queer sociology as a theoretical and methodological grounding for this study. Transnational queer sociology (see Kong 2019) is a critical decolonial theory and method that integrates queer theory with critical sociology, interrogating how Western and inter-regional concepts of homosexuality and interactions with the state shape queer identities in non-Western contexts (31–33). It explores how governance, hegemonic conceptions of sexuality and lived experience of difference (e.g. ethnicity, gender) interact to shape and mobilise queer identities. Importantly, transnational queer sociology focuses on how the flows of people and ideas within Asia are foundational for the establishment of local queer identities, recognising that Western queer culture has an impact but is subsidiary, thereby decentring whiteness (34).

Transnational queer sociology has been used in understanding gay identities across majority ethnically Chinese countries in East Asia, and is usually used to compare across different countries (31, 32, 35). This research applies these principles to defining sexualised drug use types, using comparisons between GBMSM sexual drug use cultures across Bangkok, Pattaya and Khon Kaen, Thailand.

The aim of this research is therefore to develop, informed by transnational queer sociology, a locally grounded definition of *hi-fun* compared to other sexualised drug use types practiced among GBMSM in Thailand.

We do this by introducing a typology of sexualised drug use types intended to draw boundaries around *hi-fun* versus other sexualised drug use in Thailand, and by elucidating the structural and social influences on *hi-fun* practices. This is not intended to provide a definition of all types of sexualised drug use, rather to begin the process of delineating variation and to inform further research and service provision.

## Methods

This research employed focus group discussions and supplementary interviews with GBMSM engaged in sexualised drug use in Bangkok, Pattaya and Khon Kaen, Thailand. Focus groups were selected to develop locally grounded, normative community understandings of the subject matter, allowing for the establishment of a working definition of *hi-fun* which is drawn from, and relevant to, the community. Indepth interviews were used to triangulate focus group data, and to facilitate the inclusion of GBMSM with disclosure concerns, a key issue as sexualised drug use is heavily criminalised and stigmatised in Thailand (20).

### Setting

Our study sites were selected to provide an examination of how contextual factors related to the macro (e.g. tourism, economic development, migration, inequality, legislation and the built environment) and meso (e.g. gender, race/ethnicity, socioeconomic status) environments influence sexualised drug use definitions and practices.

Bangkok was chosen as the epicentre of gay culture in Southeast Asia, attracting migrant and tourist GBMSM from other Southeast Asian, Middle Eastern, African and Western countries with gay nightlife, comparatively liberal attitudes to homosexuality and the sex industry (28, 29, 36). Pattaya offers the perspective of a major centre of gay tourism, including to engage with sex workers (37). Khon Kaen is a rapidly developing city in Isaan (a culturally distinct region in Northeastern Thailand), with a large student population and some migration from other Southeast Asian countries, but which is relatively unvisited by tourism. Khon Kaen thereby provides an ideal reference point grounded firmly in the experience of an economically emerging Thai city.

### Community involvement

This research was co-produced with our community advisory board (CAB) made up of experts from policy, clinical and community-based organisations as well as representatives from networks of people living with HIV and people who use drugs. The CAB fed into study design including data generation instruments, recruitment strategy, analysis and interpretation of our findings. All focus groups included peers in data collection.

### Recruitment and participant sampling

Eligible participants were cis or transgender men, aged 18 or older who had combined sex with a range of drugs (cocaine, GHB/GBL, ketamine, MDMA/ecstasy, methamphetamine) in the preceding 12-months. Participants were recruited through collaborating organisations with expertise in engaging and providing services to GBMSM involved in sexualised drug use. These organisations include ACTTEAM in Khon Kaen, Health and Opportunity Network in Pattaya and Institute of HIV Research and Innovation in Bangkok. Additional participants, especially non-Thai born GBMSM, were recruited through the networks of our CAB and other study investigators. Participants were firstly invited to join a focus group. If a suitable group based on language was not planned, or if they had confidentially concerns, they were invited to join an in-depth interview.

We did not use a sampling frame, however at the outset we set a target of 20% of participants be nonThai born, in order to include men from diverse sections of the GBMSM community residing in Thailand.

Participants were compensated with 1,000 THB for their time.

### Data generation

At the outset of focus groups and interview participants filled in an anonymised demographic survey capturing age, sex assigned at birth, gender/sexual identity (relying on a tool previously used in this context (38)), preferred gender/sexual identity of sexual partners, highest level of education, country of birth, membership of Thai minority ethnic group, HIV testing history and status, PrEP use, whether men had combined sex and drugs in the preceding 12-months and which drugs they used. These were later entered into a survey hosted on Qualtrics by team staff for analysis, which was done by tabulation.

We designed topic guides for focus groups and in-depth interviews drawing on extant literature (11, 13, 15, 23, 39), findings from pilot research (20), our theoretical grounding (31, 32), and CAB input (see supplementary 1). This had three sections including: 1) defining *hi-fun* and other sex with drugs; 2) influences on *hi-fun* and other sex with drugs and 3) strengths and challenges of men and communities engaged in *hi-fun* and other sex with drugs. Focus group and interview participants were asked the same general questions, although the topic guide was slightly abbreviated for in-depth interviews. See appendix 2 for full topic guides.

Two activities were carried out during focus groups. In the first, men wrote down names of substances men use to combine sex and drugs on sticky notes and placed these along a continuum based on whether they were associated with *hi-fun*, other types of sex with drugs, or both. In the second activity men brain stormed lists of strengths and challenges of *hi-fun* and other sex with drugs on flip chart, before ranking these from most to least important or pressing. The outcomes of the activities were photographed by focus group facilitators. An abbreviated version of the same topic guide was used for interviews, with less focus on the activities.

Focus groups lasted approximately 2.5 hours and interviews around 90 minutes. Three focus groups and four interviews were in Thai and two focus groups and two interviews in English. These were audio recorded, transcribed and translated to English where necessary.

### Analysis

We used thematic framework analysis (40). Two researchers (TCW and WW) familiarised themselves with all transcripts. Themes were developed based on extant literature, pilot research and emerging findings. These were mapped onto the key domains described in transnational queer sociology and discussed with the wider team. Piloting applied this coding framework to one Thai and one English language focus group. The framework was refined with additional themes added (see supplementary material 2). TCW and WW then applied this framework to all manuscripts. Following, results were shared and discussed with the project CAB, as well as representatives from all collaborating organisations, shaping nal interpretation.

### Ethical considerations

Ethical review was sought from, and granted by, the research ethics committee at the Mahidol University Faculty of Social Sciences and Humanities (ref:2024/018) as well as University College London (UCL) (ref:24583/001). All participants provided verbal recorded consent, in line with Mahidol University policies. This was done through reading an informed consent script and having participants respond to individual statements around study participation and potential risks (see supplementary material 3).

Because our study participants are uniquely vulnerable to criminalisation and police entrapment, we took the following measures to ensure confidentiality. Firstly, although we initially hoped to recruit through online sources, the risk of police infiltrating focus groups was deemed unacceptably high. We therefore pivoted only to recruitment through our networks and collaborators. Secondly, apart from participants recruited through our networks, no participant names or contact details were sought or stored. Those that were kept were deleted immediately following data generation. Thirdly, all focus groups and interviews were conducted without names. Identifying details were removed from transcripts as soon as practical. Finally, all data from our participants was held on the UCL data safe haven in the UK in order to mitigate risks of data breaches and police action.

## Results

We recruited 30 cisgender GBMSM across three study sites; 12 from Bangkok, and nine each from Khon Kaen and Pattaya. Twenty-four men took part in five focus groups, ranging from two to nine participants each. Six took part in in-depth interviews. Most (60% n = 18) had at least a bachelor’s degree, aged between 26–45 (93%, n = 28), and identified as gay (83%, n = 25). In total, 10 (33%) were living with HIV, and half were HIV negative of whom 60% were taking PrEP. The rest chose not to disclose their HIV status. Most (n = 23) had used crystal methamphetamine before/during sex in the preceding 12 months, with fewer taking other drugs (ecstasy/MDMA = 14, ketamine = 12, cocaine = 10, GHB/GBL = 5). Finally, while 23 (77%) were born in Thailand, 7 (23%) were born abroad. Table 1 presents study demographics.

Participants described three main overarching types of sexualised drug use among GBMSM in Thailand: *hi-fun* parties, sex at homepas and incidental sex with drugs. First, we briefly define and describe the three main types, before outlining how men define *hi-fun* in relation to other sexualised drug use types.

### Typology of sexualised drug use

Normative understandings of the various types of sexualised drug use practiced by GBMSM were not always clearly defined. However, for most men in our study they coalesced around the typology described below. It is important to note that for a small number of men, any combining of sex and drugs would be considered *hi-fun*. However, this was a minority view, expressed almost entirely during in-depth interviews and primarily by men not born in Thailand. The typology we describe here, and the defining characteristics following, represent normative understandings expressed by men in our study.

### Hi-fun

During focus groups, men discussed *hi-fun* at great length. *Hi-fun* was described as sexualised crystal methamphetamine (ice) use, usually with one or more men. The main goal of combining sex with ice is generally to extend the duration of the sexual session and to enhance pleasurable aspects of sex. *Hi-fun* was felt to be common in all three settings, and was potentially the most visible type of sexualised drug use practiced in Thailand.

### Sex at Homepas

*Homepas*- short for home parties and sometimes known as pool villa parties in Pattaya- are medium to large sized gatherings where GBMSM congregate to dance, socialise and take drugs, often after attending a gay event or club and usually while wearing only underwear. Drugs associated with homepas included cocaine, ecstasy/MDMA, ketamine, GHB/GBL and other stimulants. Ice was described as not included in homepas.

*Homepas* are also often organised around circuit parties or events in the gay calendar such as pride and Songkran (Thai New Year). As such, they are linked to the hi-so^1^ metropolitan gay scene, and the spacesphysical and temporal- where this intersects with regional and intercontinental tourism. Sexual activity is not a primary goal, and many attend homepas without engaging in sex, however sexualised drug use can and does occur in these settings.

*Homepas* were seen to be common in Pattaya and Bangkok because of strong links to the queer scene, which is less developed in Khon Kaen.

### Incidental sex with drugs

Men described incidental combing of sex and drugs which they would generally not consider to be hifun. This usually involved unplanned, or spontaneous combining of sex and drugs, often after leaving a bar or club or in a sex on premise venue such as a sauna. Drugs associated with incidental sex and drugs were broadly congruent with drugs associated with homepas, and included cocaine, ecstasy/MDMA, ketamine, GHB/GBL and other stimulants. Ice was not included in incidental sex with drugs, and indeed if ice was taken in these contexts, then the sexual activity would likely be considered *hi-fun*. Incidental sex with drugs was felt to be common in all three cities.

### Defining *hi-fun* compared to other types of sexualised drug use

Participants described four key criteria that helped frame whether an activity was considered *hi-fun* or another type of sexualised drug use. These were: 1) motivations and intentionality; 2) drug choice and consumption methods; 3) technology and organisation and 4) setting choice. Below we describe each criterion and elucidate how it relates to *hi-fun* in contrast to the other sexualised drug use types outlined above. We also provide an exploration of some of the unique elements of *hi-fun*. Additionally, we describe how both macro and local contexts of each of the three cities shapes practice and variation around *hi-fun*.

### Criteria 1: Motivations and intentionality

Men in our study described three primary motivations associated with sexualised drug use. These include 1) wellbeing: pleasure, intimacy and fun; 2) socio-economic: employment, status and networking and 3) queer culture: socialisation and belonging. We present each of these below.

### Wellbeing: pleasure, intimacy and fun

Across all three cities, most men described engaging in *hi-fun* as an intentional means to improve wellbeing through more intimate sex, increased sexual pleasure as well as a sense of fun and adventure. This is the primary driver for *hi-fun* and extending the sexual encounter while facilitating escapism is central to this aspiration. Indeed, *hi-fun* is usually described as a way to heighten sexual connection with other men and to feel things not possible in ones’ regular life while also reducing inhibitions and exploring new sex acts.

For me, hi-fun is another way to enhance my happiness. We use it to fulfil certain needs and bring us some joy. Each time I use it, I feel happy. […] I understand it [ice] might be considered a drug, and it is a drug, but I see its benefits in how it brings out certain feelings that I cannot express in daily life. This includes sexual feelings that I might not be able to act on in daily life. It makes me more daring and satisfies my desires, something I can’t always achieve in my normal life.

(Interview 2, Thai language, Khon Kaen)

This participant views *hi-fun* as a means to enhance happiness through escaping from ‘normal life’ and bringing sexual experiences not otherwise possible. Indeed, while many describe this sense of escapism linked to *hi-fun*, some specifically use pleasure to cope with difficult circumstances. Examples described by our participant included dealing with the emotional fallout of periods of unemployment during the COVID-19 crisis and coping with the death of a loved one.

I started using it [ice] after my mother died. I began playing alone. It started small and gradually increased. It wasn’t related to sex from the beginning. During the COVID period, after my mother died, I looked for something to hold on to, to heal my internal wounds.

(Participant 7, Thai language focus group, Pattaya)

In sex at homepas and incidental sex with drugs, increasing well-being through sexual pleasure was described a driver of participation much less frequently. This means that these types of sexualised drug use are usually seen as less intentional than *hi-fun*, and often framed as being more spontaneous.

Participant 1

I would say but it’s not often intentional, […] I suppose just going back to if you’re on a night out and then things might change or someone might offer something on a night out, and then obviously after the night out that might lead into […] into the fun part, yeah.

Facilitator 1

Hmmhmm. And is that for you… would you think that that is a type of hi-fun or is that something different?

Participant 1

I would say that it’s something different, but I wouldn’t necessarily mix [drugs with sex] intentionally for fun, if that makes sense?

(English language focus group 1, Bangkok)

This sense of spontaneity and the connections of homepas and incidental sex with drugs with social scenes more generally means that these types of sexualised drug use are more frequently seen through the lens of socialisation and queer culture, as discussed further below.

### Socioeconomic: employment, status and networks

For some men, especially those with less financial resources or those working in cities primarily economically reliant on tourism such as Pattaya, *hi-fun* can be an important strategy to increase income through sex work. While not a substantial motivation for all who engage in *hi-fun*, it can be a primary motivation for a minority. Indeed, sex work is intricately linked with *hi-fun* in all three cities, with many parties (especially highly organised events in Bangkok) having sex workers present paid for by the party hosts. Sex workers in all three cities can charge a substantial premium for *hi-fun* services, which may or may not include supplying drugs, making it a potentially attractive source of income.

Participant 6

Mainly [for me], it is for rab ngan [slang term for sex work] purposes. We work with customers, so we do it in order to earn money.

Participant 4

These jobs pay a lot, more than usual income, because you have to stay all night. It’s around 2,500–5,000 [baht per night].

(Thai language focus group, Pattaya)

Engaging in *hi-fun* can also potentially be used to seek to greater social capital through the establishment and consolidation of social networks with those of high social standing. *Hi-fun* is often seen to be a high-class activity which provides the opportunity to engage with individuals with higher social positions. In Khon Kaen especially, this appears to be a more common motivation, as *hi-fun* parties may allow access to segments of society otherwise out of reach. In contrast, a participant from Khon Kaen who identifies as having a high social position, and a *hi-fun* network made up of similar individuals, felt there was limited mixing of people from different social classes because of the risk of social discovery around combing sex with drugs, a significant concern in this midsized city.

There is no mixing of groups. Why? Because it risks their careers. They already have safe sex practices like using condoms, right? But career and reputation are beyond control. There’s no vaccine or protection for that.

(Interview 1, Thai language, Khon Kaen)

These socioeconomic motivations were not described as a major factor in engaging in incidental sex with drugs. This also was not described as a substantial motivation to engage in homepas, however as noted previously, these parties are strongly linked with the Thai *hi-so* gay scene.

### Queer culture: socialisation and belonging

Socialisation is an important but secondary motivation for *hi-fun*, as is meeting new people and feeling a sense of belonging to a scene. This can manifest as either engaging in *hi-fun* because of social pressure to conform as this type of sexualised drug use becomes more normative, or using *hi-fun* as an opportunity to socialise with others, especially when ones’ own social networks may be fragile or feels lacking.

*It’s like we’re playing as friends. We were close and came to play together. When we want to have sex, we go find it. We go different ways, but we come to play together. It’s like friendship. We’re not only focusing on sex. […] We play as part of our life, to heal the wounds and our sadness*.

(Participant 1, Thai language focus group, Pattaya)

For sex at homepas, participating in queer culture and socialising are primary motivations underpinning engagement. Homepas are seen as an extension of the social elements of gay nightlife, and sex is something that may or may not accompany that.

Participant 3

I don’t think people are meeting up and hooking up with socialising as one of- […] I imagine it will happen and you meet people and it can go from there, I think that would be more of the party scene which is where people are meeting up for that.

Facilitator 1

Yeah, the precursor to the second part.

Participant 3

Yeah. So not always… I mean some people are just, yeah, meeting up with people to go out and to party and have got no intention of going further as well but often it [sex] does [happen], yeah.

(English language focus group 1, Bangkok)

This is largely because sex at homepas as well as other incidental sex with drugs is often a by-product of socialising, and is understood within a framework of levity and fun rather than as necessarily seeking greater sexual intimacy. There is no implicit expectation that one will engage in sex, unlike in *hi-fun* contexts. However, it is important to note that in Bangkok especially, some homepas are specifically set up for individuals to engage in sex.

In Bangkok they have this culture of home parties. So in these parties, people are coming from all over, maybe from different various clubs, and then they just come together into this one hotel room or in someone’s home that already customised it to cater for a home party, so it is soundproof and all that. And in some parties, they have one dedicated room where they can have sex or there’s nothing happening in that in that party, but it’s just really a place for people to have fun, while, at the same time, meeting potential sexual partners in those settings.

(Participant 2, English language focus group 2, Bangkok)

Indeed, as described above, even within homepas which have designated spaces for sex, men generally described the motivations to attend homepas as to participate in the party scene, with sexual activity being a secondary objective. This helps to distinguish the very intentional sex that happens as part of *hi-fun* from that which occurs in homepa spaces, which was more often framed as a secondary objective. In line with this, sex at *homapas* may be more likely to include non-penetrative sex.

*As for GHB, I’ve seen it mostly in clubs, where it’s used for fun at parties like underwear party* [homepa]*. It’s a substance associated with* pai nok [sex without penetration].

(Participant 4, Thai language focus group, Bangkok)

### Criteria 2: Drugs and consumption methods

*Hi-fun*, sex at homepas and incidental sex with drugs have specific substances which are normatively associated with them. In this section we describe the substances associated with each type of sexualised drug use, and then explore the temporal, cultural and legislative forces that shape *hi-fun* drug use.

### Drugs associated with sexualised drug use types

Our participants described ice as being absolutely central, and typically foundational, to *hi-fun*. Ice is valued as a key part of this type of sexualised drug use for its stimulant properties, and because it is seen to dramatically increase sexual desire while extending the sexual session, sometimes for several days. Indeed, according to most men, without *ice* being involved the activity likely would not be considered *hi-fun*, even if involving quite similar behaviours.

I would say personally that hi-fun would exclusively be for using meth and I personally wouldn’t associate hi-fun with any other substance.

(English language focus group 1, Bangkok)

The main one [hi-fun substance] is ice - Methamphetamine. It has to be ice, almost 100% of the time.

(Interview 2, Thai language, Khon Kaen)

**Figure 1:**
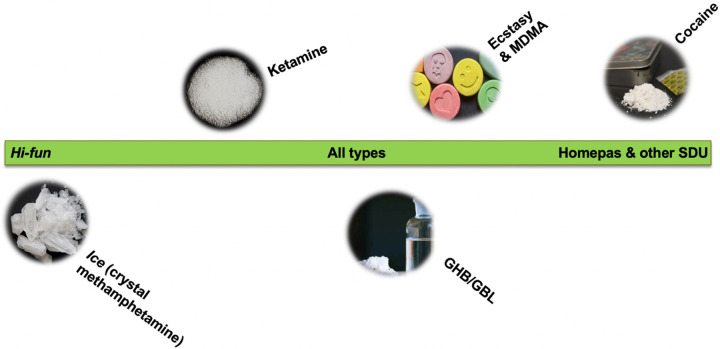
Types of drugs and associations with *hi-fun* vs homepas and other sex with drugs

While ice is absolutely central to *hi-fun*, in some circumstances other drugs might be involved. In addition to ice, GHB/GBL might be used by some men, however this is seen as uncommon in Thailand, partly because of its prohibitive cost. Ketamine might also be taken, but this would be additive to ice. MDMA and cocaine are rarely used in *hi-fun* settings. Figure 1 provides a continuum reflecting our participants’ accounts of how common drugs are in *hi-fun* versus other types of sexualised drug use settings.

In contrast, ice is rarely associated with *homepas* and incidental sex with drugs, where its use may be stigmatised or forbidden: In those situations [*homepas*] they won’t allow you to do hi [*ice*]. They will only eat cakes, snacks [*ecstasy*], and drink alcohol (Participant 8, Thai language focus group, Pattaya). Further, as ice is one of the most defining features of *hi-fun*, if it is involved in sexual activity that activity would likely fall under the *hi-fun* umbrella. Instead, *homepas* and incidental sex with drugs are associated with a much wider range of drugs. These include MDMA/ecstasy, cocaine, ketamine, and GHB/GBL.

Interviewer 1

So, meth [ice] doesn’t really exist in the context of a pool villa party [homepa], right?

Participant 8

No, there would be ecstasy, ketamine, cocaine used by specific groups.

Participant 3

Like this gay group. If it is gay-only group, there would be screening.

Interviewer 2

What kind of party is this you mean?

Participant 3

The kind that involves sex.

Interviewer 2

Do they also use meth?

Participant 3

No. But they will use ketamine, G, and ecstasy.

(Thai language focus group, Pattaya)

The drugs normatively associated with *homepas* and incidental sex with drugs are substances which are felt to be primarily social in that they increase feelings of euphoria, chattiness and are perceived and make those who take them intoxicated rather than alert, meaning they are more suitably matched to the motivations that accompany their consumption.

Ketamine is in the same category because it doesn’t stimulate alertness. Ketamine makes you intoxicated. GHB makes you intoxicated. Cocaine makes you intoxicated. Ecstasy makes you intoxicated.

(Interview 1, Thai language, Khon Kaen)

Finally, across *hi-fun*, homepas and incidental sex and drugs, there are some substances which may be used to enhance the session but are generally seen as additions to the sexualised drug use type rather than central components. This is because they can be used in many other contexts which would not fall within this typology. These include drugs to enhance the sexual session such as erectile dysfunction drugs and poppers (alkyl/amyl nitrate), as well as substances some may use to manage comedowns from stimulant drugs (cannabis, benzodiazepines). In addition, alcohol may also be used in some *homepas* and be can be a part of incidental sex and drugs.

It should be noted that a very small minority (n = 2) felt that sex including only poppers could be considered *hi-fun*. This perspective was not expressed in focus groups indicating that it is not a normative view amongst community members.

### Differing drug consumption modes across time and cultures

GBMSM in our study described differences in drug consumption modes across time and cultures related to *hi-fun*. These include changes in ice administration during *hi-fun*, as well as differences across and between the diverse cultures of GBMSM in Thailand.

Amongst our participants, smoking was perceived to be more common early in ones’ *hi-fun* career, with many participants describing their entry into *hi-fun* via this consumption method. Despite this, there is a clear consensus that injecting has become increasingly common, especially for Thai GBMSM, to the extent that it is now seen as a normative practice in *hi-fun* settings. The normalisation of injecting is felt to have been underpinned by user generated media disseminated through closed discussion groups, X (formally Twitter) and OnlyFans where injection was described as commonplace.

In Pattaya, men highlighted that periods of unemployment during the COVID-19 crisis contributed to increased ice usage and a change in their own consumption practices, and in Khon Kaen, some linked it to disruption in quality of ice during this period. In all three cities men described the process of transitioning from smoking to injection to have started in Bangkok, with GBMSM engaged in *hi-fun* in other regions of Thailand eventually following suit.

Interviewer

Participant 4 said that there was a change in using methods. What caused the change? Why aren’t people afraid of needles anymore after COVID?

Participant 2

I think maybe this [trend] came from Bangkok. It got the influence from Bangkok and Twitter. On Twitter, there is [pornographic] content using injection method.

(Thai language focus group, Khon Kaen)

Most men felt that everyone at a *hi-fun* party must use *ice* in the same way, potentially accelerating the diffusion of injection as a normative practice. Other modes of consumption, including rectal and oral administration were seen as niche practices because they are less efficient and require greater amounts of *ice* to achieve the desired effect.

Men in our study, especially those in Bangkok and Pattaya which are especially culturally diverse cities, often compared how different groups of GBMSM consumed *ice*, potentially contributing to a fracturing of *hi-fun* scenes by ethnicity. Men from other Southeast and East Asian countries are seen to be frequently involved in *hi-fun*, although preferences for administration mode appears to vary. As a general rule, individuals from China and high-income Asian countries are normatively viewed to prefer smoking. In addition, falang^2^ are assumed to inject ice less than other groups.

Participant 8

Yes, some people like the feeling of injecting and some people like the feeling of smoking.

Interviewer 1

So, in other places, they will… But now injecting is more common. Smoking is not often found. But here, they still prefer smoking.

Participant 9

It [smoking] is used by many foreigners.

(Thai language focus group, Pattaya)

Men in our study also described cultural diversity in terms of which types of drugs might be involved in *hi-fun*. This mostly centred around GHB/GBL which is seen to be very expensive and so viewed as used in *hi-fun* exclusively by wealthy, high status people, predominantly in Bangkok. Cultural groups most associated with *hi-fun* which includes these drugs are those from Hong Kong and Singapore, as well as *falang*.

### Impact of police and legislative environment on *ice* consumption

Shifts from smoking to injection have also been heavily influenced by the unique legislative and policing context of Thailand. While the legislative forces of the country impact *hi-fun* in a similar way across the three cities, the way state power is exercised differs.

Men described dramatic changes in the quality of ice over time as contributing towards the uptake of injection. These changes were linked by participants to the degree of focus of government attention on disrupting the drug market in Thailand. When attention and action were greatest, ice quality declined, and prices increased.

And the law and its enforcement also affect prices. When the laws are really strict, the prices go up. There was a period under the previous government where they were quite lenient on this issue, so prices dropped, and they remained low even until this current government. I remember when I first started using Ice, it was about 2,000–3,000 baht per gram, but now it’s much cheaper. It’s like, wow, it’s so cheap, not reflecting inflation at all.

(Interview 5, Thai language, Bangkok)

Fluctuations in price and quality linked to enforcement action were also perceived to have led to increases in injection to ensure optimal use of *ice* when drugs were of potentially inconsistent quality.

A further pressure encouraging injection drug use is the legislative environment itself. The legal system in Thailand sets limits on the amount of drugs in ones’ possession that is considered for personal use versus trafficking, with individuals possessing small amounts meant to be spared imprisonment. Because of this, there are high incentives for individuals to carry only very small quantities of drugs. This increases the attractiveness of injecting rather than smoking ice, as it is a much more efficient mechanism of consumption and drug effects are perceived to last for longer.

In all three cities men had substantial concern about police entrapment, arrest and prosecution which contributed to an environment in which injection became increasingly normative. Because injection equipment is much easier to conceal and dispose of than the pipes used to smoke ice, potential for contact with the police further incentivises injection.

Because…but about a year or years ago, more people started to use injections. […] what I’ve heard is that there’s also the issue of equipment. Storage and disposal of things like this will be easier. (Interview 4, Thai language, Bangkok)

This police action is enacted in different ways across the three cities. In Bangkok, police checkpoints mean that ease of concealment and disposal is a key consideration in organising *hi-fun*. In addition, police were felt to solicit bribes from those caught carrying substances at checkpoints. Men therefore prioritised carrying smaller amounts under the expectation that these bribes might be less. In Khon Kaen, where *hi-fun* often takes place in hotels where the risk of discovery is higher, the need to ensure that materials can be disposed of quickly should the police arrive is amplified. This is further underpinned by concern about the noise made by water pipes while smoking ice. Finally, in recent years police action closed many shops in Khon Kaen selling smoking equipment making injection a more accessible option.

Participant 2

There were shops selling such [smoking] equipment in the past, but they went bankrupt and the police took them down.

Interviewer

And the equipment become hard to find.

Participant 4

It’s difficult to carry around and costs money. Injection involves only needles, which can be thrown away after use. However, for the glass tube or smoking method, you have to carry the equipment with you all the time.

Participant 2

It also must be assembled.

Participant 5

And again, when pulling [smoking], there will be a knock, knock, knock sound. […] It sounds like a longtail boat.

(Thai language focus group, Khon Kaen)

In Pattaya, GBMSM engaged in *hi-fun* did not describe substantial direct impacts from police action on *hi-fun* drug administration mode. However, participants noted that relationships with the police are perilous, with some officers felt to be involved in ice distribution and potentially as *hi-fun* participants, compounding GBMSM’s vulnerability to extortion and arrest.

### Criteria 3: Technology and organisation

Men described the centrality of apps and social media to *hi-fun*. In this section we describe how apps are vital in arranging *hi-fun* sessions, and how social media acts as a site for cultural expression. We also outline the potentially more limited role of technology in incidental sex with drugs and sex at homepas. Finally, we explore men’s descriptions of establishing *hi-fun* networks rooted from initial contacts in online spaces.

### Apps, social media and cultural expression

As in other settings, geo-location social sexual networking applications (apps) are the key way men arrange *hi-fun* sessions. Men use these apps at particular times, often at night, to identify potential *hi-fun* partners and organise sessions. The apps most often used in the three cities of this study are Hornet and Grindr: Hornet is perceived to be most useful for searching wider geographical areas, whereas Grindr is often more associated with those specifically seeking out *hi-fun*.

Participant 1

Yes. Orange app [Hornet] as well as the mask app [Grindr]. But people in the mask app tend to have more much more drugs than the orange app.

Participant 4

Mostly, people from the yellow app [Grindr] have 100% more substances. But the orange app [Hornet] allows you to search more widely than the yellow app, which focuses more on nearby people. It is easier to find in the yellow app than in the orange app.

(Thai language focus group, Bangkok)

Men described that *hi-fun* parties often start with men using *ice* either on their own or with one or two other men, with additional participants invited through the apps. Initial discussions may involve negotiations about drug supply, payment for drugs, details of other participants and potentially include video calls to ensure prospective participants are not police.

Closed discussion groups on social messaging apps such as LINE and WhatsApp are also used to meet partners. However, these are more often seen as a way to discuss *hi-fun* related activities and share pornography, including user generated content. This is at least in part because of concerns around scams and potential police infiltration.

LINE groups require an invitation from a friend. You can’t just join them randomly. I’ve never joined one myself. Also, these platforms… let’s put it this way, they’re not as reliable as they used to be. Four or five years ago, these platforms were great for finding sex, whether regular sex or hi-fun. But recently, with so many news reports of scams and misrepresentations, people have started to pull back. They might browse but are less likely to arrange meetups. […]

(Interview 1, Thai language, Khon Kaen)

In contrast to *hi-fun*, homepas and incidental sex with drugs are seen as an extension of the gay scene and/or partying, and are therefore not as firmly linked to apps. For homepas especially, these appear to be organised primarily within networks of friends and extended friendship groups as opposed to sexual networks, although social media may be used in engaging a wider audience for these events. Incidental sex with drugs is more normatively associated with meeting people in person, especially at bars, clubs and sex on premise venues.

### Networks: open and closed

Men in our study discussed developing *hi-fun* networks over time. These were rooted primarily from partners they met on, or parties they attended through, apps. *Hi-fun* networks are perceived to be divided by ethnicity in Bangkok and potentially by social class in Khon Kaen. In Pattaya, networks appear to be very mixed, likely reflecting that the city has a relatively modest population which welcomes very high number of GBMSM tourists who come to engage in *hi-fun*, as well as GBMSM residents from other countries.

Establishing these networks is also a strategy employed by many to avoid potential interactions with law enforcement, as the police are seen to frequently pose as men hosting *hi-fun* parties in order to entrap and extort GBMSM who use drugs. Indeed, trusted networks become a key component of managing this risk.

*If I were to use dating apps, it would probably be for sober sex. I don’t look for people on dating apps anymore because you never know if they might be scammers or undercover police. I feel it’s better to stick with the same people within my circle. It might not be a lot of people or very diverse, but at least it’s the same familiar people, and I’m pretty sure they’re not going to turn out to be undercover cops or anything like that*.

(Interview 5, Thai language, Bangkok)

In Bangkok, men also described that tourists from other Southeast/East Asian countries utilised sex workers and clandestine agencies to pre-organise *hi-fun* parties, often from their home countries before travelling. How these operate is currently unknown. When discussing whether *hi-fun* scenes were mixed by ethnicity, one participant said the following:

Interviewer

That means there aren’t many settings where Westerners, Thais, and Taiwanese are together in the same group or party. Is that correct?

Participant 4

There are, but it’s hard to access. It’s more like an agency. From what I have seen, there will be providers.

Interviewer

So, there is an agency to provide like this?

Participant 4

Yes.

Interviewer

Well, they would send a request from their country that…

Participant 4

They know each other, like friends or something like that. They all know that they want to come to Thailand and want to come to play.

(Thai language focus group, Bangkok)

### Criteria 4: Sexualised drug use setting choice

How and where various types of sexualised drug use took place was often mediated by the built environment and its role in potential for discovery and subsequent possible criminalisation. In *hi-fun* settings, risks also includes the potential from violence from other men. Both *hi-fun* and homepas are normatively associated with private spaces, whereas incidental sex with drugs is linked to a potentially broader range of venues. In this section we outline how these considerations play out when considering *hi-fun*, homepas and incidental sex with drugs in both private and public spaces, paying attention to the role of the built environment.

### Private spaces, varying risks

According to our participants, the majority of sexualised drug use in Thailand takes place in private settings, including houses, condos, apartments, hotels and short-term rented accommodation. Both *hi-fun* and *homepas* are normatively associated with private spaces, whereas incidental sex with drugs is linked to a potentially broader range of venues.

Having *hi-fun* gatherings in apartments and houses is seen to be relatively uncommon in Khon Kaen and Pattaya because these settings lack anonymity, with high potential for gossip from neighbours. In contrast, engaging in incidental sex with drugs at home or in hotels was generally seen as unproblematic because these gatherings were shorter in duration and usually included fewer men than *hi-fun* parties or homepas.

In Bangkok, where people commonly live in larger condominium buildings, having *hi-fun* at an apartment can be more desirable. This is because condo buildings allow for greater security against the risk of criminalisation as police must first bypass building staff to gain entry, which is felt to be uncommon. Men also described how homepas also sometimes take place in domestic settings, especially where men have customised condos with soundproofing and have the benefits of barriers to police access. Houses which don’t have security are seen to have additional risk around hosting *hi-fun* (and potentially homepas) because of easy access by the police.

Interviewee

Mostly, it [hi-fun] would be at home, or in a hotel. But it’s usually more in a condo because it’s safer […]

Interviewer

And why isn’t a house safe?

Interviewee

Because if the police get involved, they can get in easily. In a condo, there’s still security to deal with first—they’d have to ask questions.

(Thai Language focus group, Bangkok)

In Khon Kaen and Pattaya *hi-fun* is often practiced in hotels, primarily in response to concerns around domestic privacy in houses. When considering *hi-fun* in a hotel, men often take great care to avoid discovery. This includes strategies such as not making noise, injecting ice rather than smoking to minimise sound and smell, and leaving periodically during the day to avoid suspicion from hotel staff.

Yes, mostly we stay in the room during that time. If it’s me, if I’m staying at a hotel for two nights, I go out during the day to buy something to eat to show that everything is normal. But at night, we run the drugs all night, until 5 or 6 in the morning, starting from the evening.

(Interview 3, Thai language, Khon Kaen).

Some also hire standalone villas in resorts, as these are felt to be particularly suitable because they are more soundproof and have enough space for larger groups. Finally, some choose hotels which require key cards to access guest floors to further reduce the risk from police; these however can pose logistical challenges when inviting additional participants.

Despite the term homepa being an abbreviation of ‘home party’, hotels in Bangkok and short let rented pool villas in Pattaya are locations normatively associated with homepas, and felt to potentially be a primary site where these types of parties take place.

### Public spaces, increased risks

Saunas and public sex environments are rarely associated with *hi-fun* outside of user generated pornography disseminated in online spaces. *Homepas* are so normatively linked to the private sphere that an activity taking place outside of this would not qualify. Indeed, men primarily make use of these venues for *hi-fun* or incidental sex with drugs if there are no other options available, or if it is a component of a particular sexual fetish. This is because these settings are seen to come with substantial downsides with regards to violence from other men, from police as well as additional environmental risks. When asked where *hi-fun* takes place, one participant described:

Mostly in condos, rooms [apartments], houses. Sometimes, from what I have seen in [pornography on] Twitter, it can be outside, like at beachfronts, railroad tracks, or dark and abandoned places like houses and buildings. I’ve seen a lot of them. I tried it once, but I was scared because there were so many mosquitoes.

(Participant 8, Thai language focus group, Pattaya)

In saunas, beyond the risks of transporting the drugs and being caught by staff, there are additional downsides to having *hi-fun* as ensuring sanitation around injecting ice is challenging and smoking is generally not possible.

*I would feel that if someone invited me to inject in a sauna, I wouldn’t go because I would wonder how they manage cleanliness, sterilization, and hygiene. Where are you going to dispose of these things* [needles]? *Are you going to carry the drugs from home to the sauna? The risks just keep increasing*.

(Interview 5, Thai language, Bangkok)

However, some men report how saunas can be useful as a venue to engage in sex following consuming drugs at bars and clubs.

## Discussion

In our study with 30 cis-gender GBMSM engaged in sexualised drug use across three cities in Thailand, we found that men primarily divided sexualised drug use into three main types: *hi-fun*, sex at homepas, and incidental sex with drugs. Men mostly conceptualised and defined *hi-fun* based on 4 key criteria: 1) motivations and intentionality; 2) drugs and consumption modes 3) technology and organisation and 4) setting choice. Although sex at homepas and incidental sex with drugs share some commonalities across some of these areas, teasing out these tensions allows a more nuanced understanding of how men view the range of ways sex and drugs can be combined, as well as the meanings and the motivations underpinning these practices.

We propose a definition of *hi-fun* for use in further research and service provision in Thailand. According to our results, *hi-fun* in Thailand is normatively understood as the intentional combining of sex and crystal methamphetamine (ice) to enhance intimacy and pleasure with one or more other man/men, facilitated by technology and usually in a private setting. This definition, drawn from community understandings of *hi-fun*, will likely be applicable for GBMSM across the country, although some regional variations are likely. It should also be noted that among men who engaged in transactional sex, the primary *hi-fun* motivation was financial while increasing pleasure the primary goal of the client. A separate definition of transactional *hi-fun* should be developed with men who sell sex and provide *hi-fun* services. This group should also be prioritised in future research, especially in Southeast Asia (22). We also recognise that other populations in Southeast Asia, especially transgender women, practice sexualised drug use in ways similar to *hi-fun* (41), future research should be conducted with this group to understand the unique formations this may take.

This research is, to our knowledge, among the first academic explorations of homepas. Indeed, the only publication we were able to find discussing homepas in the academic press was an article attributing the creation of the term homepa to an incident in Taipei in January 2004 where 92 party participants were arrested while using drugs and having sex (42). Despite this lack of academic focus, content from podcasts and YouTube videos indicates that homepas are common in major gay metropolitan centres in East and Southeast Asia (e.g. Bangkok, Hong Kong, Taipei) (43, 44). Much more exploration is required into homepa cultures, as well as the unique pleasures and potential risks associated with this type of socialisation.

### Intentionality and differences between sexualised drug use types

As described, *hi-fun*, homepas and incidental sex with drugs were generally considered to be three unique ways that men combine sex and drugs. Despite this, was there some ambiguity between men on the exact boundaries between these. It may be most useful to consider these sexualised drug use types as existing on a spectrum of intentionality. In *hi-fun*, combining sex with ice is the primary activity and therefore profoundly intentional. In homepas the primary goal is socialising and participating in queer culture, meaning that the sexual aspects tend to be less deliberate. In incidental sex with drugs, sexual activity is often conceptualised as an extension of a night out, again de-emphasising intentionality surrounding the sexual elements of this behaviour.

The role of intentionality is further highlighted by the drugs involved: ice is foundational to *hi-fun* and viewed as the most appropriate drug because it is seen to stimulate alertness and sexual desire. In contrast, cocaine, ecstasy/MDMA, ketamine and GHB/GBL are felt to be intoxicating drugs, increasing euphoria and facilitating socialisation, meaning that these are more appropriate for homepas and incidental sex with drugs where the primary motivations trend towards the social. While the exact role of technology in facilitating homepas remains underexplored, it appears hosts make use of social media (especially Instagram) in vetting and inviting participants (43, 44). *Hi-fun* organisation in contrast makes use of apps and sexual networks in a perhaps more intentional way than are used in incidental sex with drugs and to organise homepas.

### Structural and individual level influences on *hi-fun*

Through the lens of transnational queer sociology (31, 32), we found key influences shaping *hi-fun* across the three cities. The legislative environment and patterns of policing in Thailand have the most outsized impact and heavily influenced *hi-fun* practices and organisation, requiring a unique response. We found this was enacted through multiple pressures drawn from interactions with the state which varied by locale, but all of which incentivised injecting ice rather than smoking to reduce risks associated with criminalisation. This paper therefore contributes to a body of literature on how legislative environments can increase injection drug use (45), and extends an emerging body of work from Southeast Asia detailing the unique risks of the intensive criminalisation of drugs in the region and how interactions with state actors shape sexualised drug use practices (15, 20, 46).

This study also contributes to an emerging literature on the role of the built environment in shaping drug use practices (47, 48). In all three cities, we found that *hi-fun* locations are determined by navigating risks around criminalisation and potential violence from other men, while also considering the realities of urban geographies.

In terms of individual level factors, *hi-fun* is most frequently motivated by a desire to use pleasure and intimacy to increase well-being. For some men escapism from difficult circumstances in life is also a motivation, echoing research from the region (39, 49). This appeared to be especially pronounced during the economic disruption of the COVID-19 crisis in Pattaya, when many of our participants lost their employment, a situation which has largely been ameliorated since the lifting of restrictions on tourism.

Individual socioeconomic status also has an impact on how *hi-fun* is practiced. Motivations for engaging in *hi-fun* for some men- including those not engaged in sex work- can include the opportunity to establish and build social networks with individuals of higher socioeconomic status. This may contribute to unequal power dynamics which threaten autonomy and agency in *hi-fun* settings. Further, socioeconomic position also impacts on which drugs are involved in *hi-fun*. While ice is seen to be inexpensive and widely used, drugs associated with chemsex in other settings are more rarely included, partly because of prohibitive costs. This may mean that individuals engaging in *hi-fun* with unfamiliar drugs have less knowledge around how to consume them safely, a particular issue with GHB/GBL.

### Recommendations for policy and practice

Many *hi-fun* harms, as expressed by participants in this study and previous research (20), emerged from the legislative environment (criminalisation) and police interactions (extortion and abuse). These interlinked issues also incentivised injection, potentially leading to increases in blood borne virus transmission. Further, there are also potential harms related to limited knowledge of less commonly used drugs, a possible lack of ‘correct’ knowledge for those used frequently. Encouragingly, other recent research has found that online communities in Thailand often provide mutual care by and for those engaged in *hi-fun*, presenting additional supportive opportunities (20).

Therefore, harm reduction service provision and policy in Thailand will need to go beyond issues of health and focus on well-being more broadly. Components critical to reduce potential harms in this population include:

- Legal services to prevent and avoid abusive incidents from the police. These could include legal/justice system navigation as part of routine service provision, as well as online.
- Drug use literacy appears to be lacking. Comprehensive information about drugs should be made publicly available from trusted sources and standardised/tailored to local contexts. Online spaces are likely especially suitable avenues for harm reduction interventions.
- As injection of crystal methamphetamine is becoming increasingly common, needle and syringe provision must be an essential part of harm reduction service provision, including to GBMSM who use stimulant drugs.
- At a structural level, drug law and policy reforms reducing legislative harms are key, as well as policy implementation standardisation.

In order to support the effective implementation of the above initiatives, additional research is required amongst the family and friends of GBMSM who use drugs, as well as diverse professional stakeholders in Thailand including policy makers, police officers and healthcare providers.

### Limitations

This study represents one of the first explorations delineating different sexualised drug use types among GBMSM in Southeast Asia, and indeed more broadly. We note some limitations. Firstly, because of the risks of criminalisation amongst our participants, we relied on a narrow set of recruitment tools, mainly engaging men currently accessing sexual health and harm reduction services and those in our social networks. Because of this, the views of those not engaged with services are absent. Secondly, our participants- especially those in Bangkok- were highly educated, with nearly two thirds to University level. This means that the most marginalised were likely not engaged in our study. Fourthly, although 10% of our sample were GBMSM from other Southeast Asian countries, none were from the less developed countries immediately bordering Thailand, a group potentially more likely to experience vulnerability related to having an insecure immigration status. Finally, this exploratory study was designed to delineate boundaries around *hi-fun* compared to other types of sexualised drug use. It was not intended to provide an in-depth exploration of the cultures and practices surrounding each sexualised drug use type identified. Future research investigating the unique organisations, pleasures and potential risks linked with homepas especially is critical.

## Conclusions

GBMSM in Thailand primarily divided sexualised drug use into three main types: *hi-fun*, sex at homepas, and incidental sex with drugs. These were conceptually delineated based on 4 key criteria: 1) motivations and intentionality; 2) drugs and consumption modes 3) technology and organisation and 4) setting choice. Based on our results, *hi-fun* in Thailand can be defined as the intentional combining of sex and crystal methamphetamine (ice) to enhance intimacy and pleasure with one or more other man/men, facilitated by technology and usually in a private setting. This definition will be useful for those supporting GBMSM in Thailand through public policy, research and service provision.

## Supplementary Files

This is a list of supplementary files associated with this preprint. Click to download.

- Supplementary1.Topicguides.docx
- Supplementary2.Thematicframework.docx
- Supplementary3.Consentdocuments.docx

## Figures and Tables

**Table 1:** Participant demographics

Age	n = 30
18–25	1
26–35	16
36–45	12
46+	1
**Sexual orientation**	
Gay	25
Bisexual	5
**Born in Thailand**	
Yes	23
No	7
**HIV status**	
Negative	6
Negative taking PrEP	9
Diagnosed with HIV	10
Prefer not to say	5
**Educational qualification** *	
High	18
Medium	3
Low	9
**Location**	
Bangkok	12
Khon Kaen	9
Pattaya	9
**Drugs taken in prior 12 months**	
Cocaine	10
Ecstasy/MDMA	14
GHB/GBL	5
Ketamine	12
Methamphetamine	23

* High = Bachelor’s degree or higher; medium = completed secondary school; low = below secondary school

## Data Availability

Due to their personally identifiable material and the risk of criminalisation to our participants, underlying data will not be made publicly available. Reasonable requests will be considered on a case-by-case basis and can be made to the corresponding author.

## Footnotes

1. Thai abbreviation of ‘high-society’.

2. Colloquial Thai term for Westerners, usually also denoting that the person is white.

## Abbreviations

CAB: Community Advisory Board
GBMSM: Gay, bisexual and other men who have sex with men
GHB/GBL: gamma hydroxybutyrate/gamma butyrolactone
MDMA − 3,4: methylenedioxymethamphetamine
PrEP: HIV pre–exposure prophylaxis
SDU: Sexualised drug use
